# The pathogenic actinobacterium *Rhodococcus equi*: what's in a name?

**DOI:** 10.1111/mmi.14267

**Published:** 2019-06-17

**Authors:** José A. Vázquez‐Boland, Wim G. Meijer

**Affiliations:** ^1^ Microbial Pathogenesis Group, Edinburgh Medical School (Biomedical Sciences – Infection Medicine) University of Edinburgh Chancellor's Building, Little France campus Edinburgh EH16 4SB UK; ^2^ UCD School of Biomolecular and Biomedical Science University College Dublin Dublin 4 Ireland

## Abstract

*Rhodococcus equi* is the only recognized animal pathogenic species within an extended genus of metabolically versatile *Actinobacteria* of considerable biotechnological interest. Best known as a horse pathogen, *R. equi* is commonly isolated from other animal species, particularly pigs and ruminants, and causes severe opportunistic infections in people. As typical in the rhodococci, *R. equi* niche specialization is extrachromosomally determined, via a conjugative virulence plasmid that promotes intramacrophage survival. Progress in the molecular understanding of *R. equi* and its recent rise as a novel paradigm of multihost adaptation has been accompanied by an unusual nomenclatural instability, with a confusing succession of names: "*Prescottia equi*", "*Prescotella equi*", *Corynebacterium hoagii* and *Rhodococcus hoagii*. This article reviews current advances in the genomics, biology and virulence of this pathogenic actinobacterium with a unique mechanism of plasmid‐transferable animal host tropism. It also discusses the taxonomic and nomenclatural issues around *R. equi* in the light of recent phylogenomic evidence that confirms its membership as a *bona fide Rhodococcus*.

## Graphical abstract

Vázquez‐Boland and Meijer review the genomics, biology and virulence of *Rhodococcus equi*, a pathogenic actinobacterium that displays plasmid‐driven animal host tropism. Recent problems regarding the taxonomy and nomenclature of *R. equi* are also discussed.

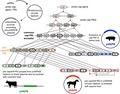

## *Rhodococcus equi*

**Figure 1:**
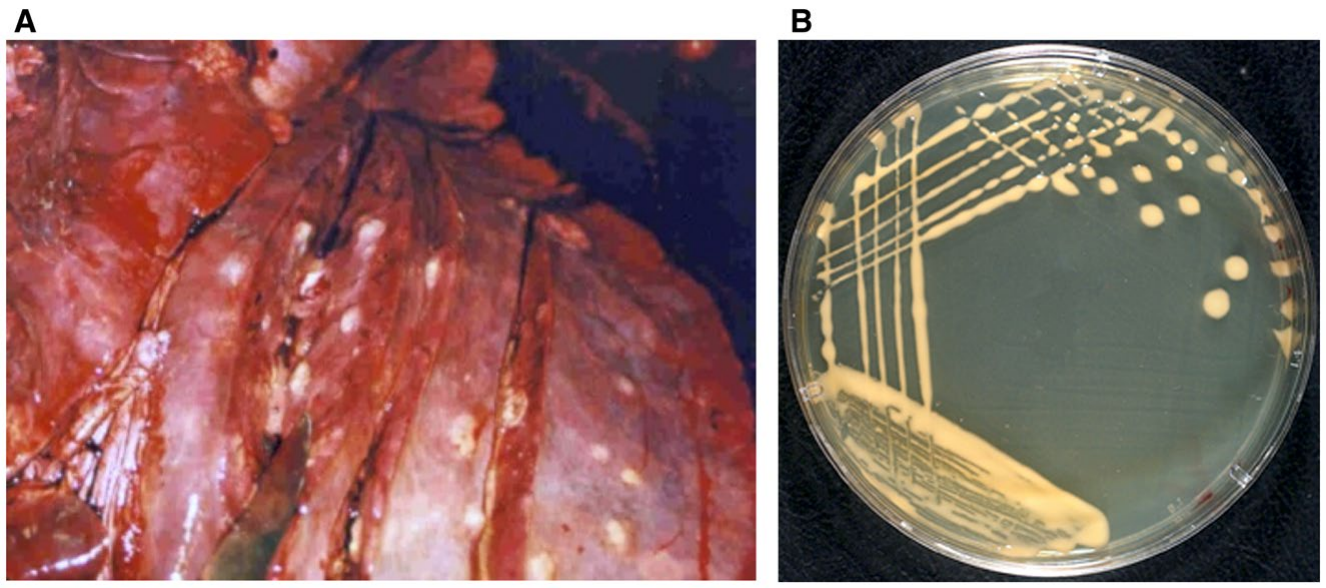
*R. equi* lung infection and colony morphology. A. Purulent bronchopneumonia in foal with multifocal abscesses. Courtesy of Dr. U. Fogarty, Irish Equine Centre. B. Typical mucous appearance of *R. equi* colonies (LB agar incubated at 30°C for 48 h).

*Rhodococcus equi* is a high‐G+C Gram‐positive, facultative intracellular coccobacillus that parasitizes macrophages, causing pulmonary and extrapulmonary pyogranulomatous infections in different animal species and people (Prescott, 1991; von Bargen and Haas, 2009; Vazquez‐Boland *et al.*, 2013). Since its discovery in 1923 by H. Magnusson in Sweden as the causative agent of purulent bronchopneumonic disease in foals (Magnusson, 1923) (Fig. 1A), *R. equi* is well known in veterinary medicine as a major horse pathogen (Muscatello *et al.*, 2007; Giguere *et al.*, 2011). In humans, it mostly affects immunocompromised individuals, notably HIV‐infected patients, where the infection resembles pulmonary tuberculosis (Yamshchikov *et al.*, 2010). *R. equi* is ubiquitous in soil, multiplies in herbivore manure and the large intestine, and spreads in the farm habitat presumably via faecal‐oral cycling (Muscatello *et al.*, 2007; Vazquez‐Boland *et al.*, 2013). Lung infections are likely contracted through inhalation of airborne dust particles carrying *R. equi* (Muscatello *et al.*, 2006; Cohen *et al.*, 2008; Petry *et al.*, 2017).

Initially named *Corynebacterium equi* by H. Magnusson himself, *R. equi* was transferred in 1977 to the genus *Rhodococcus* (Goodfellow and Alderson, 1977), currently within the *Nocardiaceae* in the order *Corynebacteriales*. *R. equi* shares a protective mycolic acid‐containing cell envelope with other members of this group of *Actinobacteria*. Like other rhodococci, it is strictly aerobic and non‐motile, forms orange‐salmon pigmented colonies (Fig. 1B) and shows coccus‐to‐rod or (occasionally) branched cell shape transition (Jones and Goodfellow, 2012). The genus *Rhodococcus* comprises at least 57 species and an ever‐growing number of unclassified isolates. Many of these are of considerable significance for the environmental, pharmaceutical and energy sectors owing to their versatile catabolic and biocatalytic properties (van der Geize and Dijkhuizen, 2004). Two rhodococcal species are recognized as pathogenic, *Rhodococcus fascians*, which causes leafy gall in plants (Stes *et al.*, 2013), and *R. equi*.

## *R. equi* genome

**Figure 2:**
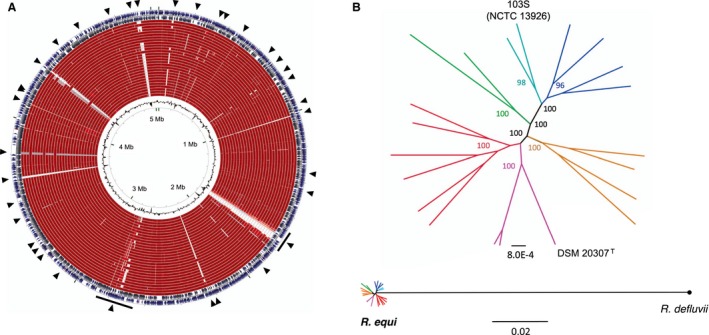
Genomic relatedness of *R. equi* isolates. Modified from Anastasi *et al.* (2016). A. Circular diagram of *R. equi* 103S (= NCTC 13926, DSM 104936) chromosome (5.02‐Mpb, outer ring with forward and reverse strands) compared to draft genomes of representative isolates from different sources and genetic lineages (inner rings). BLASTn alignments, red colour indicates > 98% sequence identity. HGT regions in 103S (arrow heads) coincide with gaps in the DNA alignments, indicating they are strain‐specific or less conserved**.** B. Core‐genome maximum‐likelihood phylogeny of *R. equi* isolates in A. Top, unrooted tree; reference genome isolate 103S and type strain of the species are indicated. Bottom, tree rooted with the closely related species, *R defluvii* Ca11^T^. The star‐like topology in the early branchings of the *R. equi* lineage suggests that the species’ diversification occurred through rapid clonal radiation from the common progenitor. See also Fig. 5.

The only complete and manually curated genome sequence available for *R. equi* is from strain 103S (= NCTC 13926 = DSM 104936), a prototypic equine clinical isolate (Letek *et al.*, 2010). The reference 103S genome (NCBI RefSeq NC_014659.1, accession FN563149.1) consists of a circular chromosome of 5.04 Mbp with 4,598 predicted genes and a G+C content of 68.8% (Fig. 2A). A second key genome component is the virulence plasmid, which carries the *vap* pathogenicity island (PAI) (Takai *et al.*, 2000). In 103S, it is a circular plasmid of 80.6 kb designated pVAPA1037 (reference sequence accession AM947677) (Letek *et al.*, 2008). The *R. equi* chromosome appears to be genetically stable, as indicated by the rarity of DNA mobility genes or insertion sequences (Letek *et al.*, 2010) and absence of significant recombination (Anastasi *et al.*, 2016). A small number of pseudogenes (14 in 103S, most in horizontally acquired regions) suggests that it is under strong selection.

Comparative genomic analyses show that *R. equi* is genetically homogeneous and clonal, with a large core genome equivalent to ≈80% of an isolate's gene content. It is a well‐defined taxon with an Average Nucleotide Identity (ANI) of 99.13% and 100% 16S rDNA sequence identity. In a core‐genome phylogenomic tree, *R. equi* isolates radiate at a short genetic distance from each other (0.001–0.002 substitutions per site) (Fig. 2B), consistent with a relatively recent evolutionary origin and a rapid clonal diversification from the common progenitor.

Like many other bacteria, *R. equi* has an open pangenome. Although non‐core genes only represent ≈20% of each strain's gene content, a significant proportion of the accessory genome (60%) is only present in one or two isolates, accounting for the intraspecific variability. *R. equi* genome evolution is primarily driven by gene gain/loss processes, with a significant contribution of horizontal gene transfer (HGT) events (Letek *et al.*, 2010; Anastasi *et al.*, 2016) (Fig. 2A). Phages are abundant in *R. equi* (Summer *et al.*, 2011; Petrovski *et al.*, 2013; Salifu *et al.*, 2013) and probably play an important role in HGT‐driven genome plasticity.

## Core *R. equi* traits

Comparative genomic studies confirmed that most traits predicted to be important for *R. equi* biology and niche adaptation belong to the core genome (Anastasi *et al.*, 2016). This includes all putative pathogenicity determinants identified in the 103S chromosome, notably a number of mycobacterial virulence gene homologs (Letek *et al*., 2010). Genes involved in tolerance to desiccation and oxidative stress, presumably important for survival in dry soil and transmission via aerosolized dust, also belong to the core genome. There is also a conserved intrinsic resistome with several putative β‐lactamases, aminoglycoside phosphotransferases and multidrug efflux pumps. These probably contribute to the variable susceptibility reported for *R. equi* to diverse antimicrobials, as observed, for example, with β‐lactams and quinolones (Nordmann and Ronco, 1992; Mascellino *et al.*, 1994; Soriano *et al.*, 1998; Makrai *et al.*, 2000; Jacks *et al.*, 2003; Letek *et al.*, 2010; Yamshchikov *et al.*, 2010).

A distinctive characteristic of *R. equi* is a complete absence of phospho*enol*pyruvate:carbohydrate transport system (PTS) components, consistent with an eminently asaccharolytic metabolism. Among the rhodococci, only its close relative *Rhodococcus defluvii* (Kämpfer *et al.*, 2014) also lacks a PTS sugar transport system (Anastasi *et al.*, 2016), indicative of specific gene loss in the common ancestor of the *R. equi*‐*R. defluvii* sublineage (see below Fig. 5). The absence of PTS homologues is rather unique within the *Actinobacteria*; among the few examples are *Mycobacterium tuberculosis*, also a parasite of macrophages, and the obligate intracellular pathogen *Tropheryma whipplei* (Barabote and Saier, 2005; Letek *et al.*, 2010). This suggests that loss of PTS sugar transport might be associated with adaptation to intracellular parasitism in this bacterial group.

Recently, genes encoding putative non‐PTS transporters for glucose (GlcP) and ribose (RbsCB) have been identified in the *R. equi* core genome. Both permeases seem to be functional, although utilization by *R. equi* 103S of ribose and, particularly, glucose is inefficient (and variable for the latter) compared to preferred carbon sources such as lactate or acetate (Letek *et al.*, 2010; Anastasi *et al.*, 2016). Since *R. equi* assimilates carbon principally via short‐chain organic acids and lipid catabolism, these two sugar transporters might act as occasional ‘nutritional fitness’ enhancers in specific habitats.

In addition to monocarboxylate and dicarboxylate transporters, the *R. equi* core genome encodes an extensive array of lipases (both secreted and intracellular) and β‐oxidation enzymes. There are also three complete *mce* (‘mammalian cell entry’) systems, which form channel mechanisms specialized in lipid transport (Ekiert *et al.*, 2017), for example, cholesterol (Mohn *et al.*, 2008; Pandey and Sassetti, 2008). Similar to *M. tuberculosis* (Muñoz‐Elias and McKinney, 2005), *R. equi* virulence requires the glyoxylate shunt enzyme isocitrate lyase (ICL) (Wall *et al.*, 2005). ICL mediates the diversion of TCA cycle intermediates for gluconeogenesis and carbohydrate biosynthesis from acetylCoA generated through fatty acid β‐oxidation or acetate oxidation. This indicates that, like the tubercle bacillus, *R. equi* utilizes lipids as *in vivo* growth substrate. Interestingly, although not a fermentative organism, *R. equi* possesses a putative bifunctional D‐xylulose 5‐phosphate (X5P)/fructose 6‐phosphate (F6P) phosphoketolase (Xfp) (Meile *et al.*, 2001). This enzyme may provide flexibility in carbon and energy metabolism by converting pentose phosphate pathway (PPP) and glycolytic intermediates into acetyl phosphate (and acetate/acetyl‐CoA) (Ingram‐Smith *et al.*, 2006).

*R. equi* appears to be particularly well adapted for growth on exogenous L‐lactate, with a dedicated transporter (LldP) and determinants for its conversion into acetate, either directly (L‐lactate monooxygenase) or via pyruvate (*lutABC* operon [Chai *et al.*, 2009]) combined with pyruvate decarboxylation via pyruvate dehydrogenase [cytochrome]) (Letek *et al.*, 2010). Moreover, *R. equi* has denitrification capacity, with a NarK nitrate/nitrite transporter, NarGHIJ nitrate reductase and a NirBD nitrite reductase. It also has the ability to grow on urea as the sole nitrogen source through the action of a urease and an ATP‐dependent urea carboxylase (Letek *et al.*, 2010; Anastasi *et al.*, 2016).

Another core feature is the disruption of the *thiCD* locus by an HGT island, rendering *R. equi* auxotrophic to thiamin (Letek *et al.*, 2010; Anastasi *et al.*, 2016). Apart from this, *R. equi* is otherwise not nutritionally demanding and can grow vigorously in the presence of just inorganic N (e.g. in the form of ammonium chloride) and an organic acid as a carbon source. Together with its alkalophily (optimal growth between pH 8.5 and 10) (Letek *et al.*, 2010), the nutritional and metabolic profile of *R. equi* may confer a competitive advantage in manure and the large intestine, its natural reservoirs, where there is an easy access to microbiota‐derived thiamine and lactate/short‐chain fatty acid fermentation products (Letek *et al.*, 2010; Anastasi *et al.*, 2016). Via a NiFe‐type hydrogenase, *R. equi* has the potential to utilize H_2_, released through microbial metabolic activity, potentially contributing to survival in the intestinal habitat.

## *R. equi* illuminates rhodococcal genome evolution

*R. equi* possesses a more compact genome compared to environmental rhodococci, exemplified by *Rhodococcus erythropolis* PR4 (6.52 Mb) and, particularly, *Rhodococcus jostii* RHA1 (7.80 Mb) or *Rhodococcus opacus* B4 (7.25 Mb), for which complete genomes are also available (McLeod *et al.*, 2006; http://www.nite.go.jp/index-e.html). Analysis of gene duplication and HGT events, together with the slow rate of gene decay in the 103S chromosome, indicated that the genome size differences are due to genome expansion in the environmental species rather than genome contraction in *R. equi* (Letek *et al.*, 2010). Genome expansion is due to amplification of paralogous families and acquisition of HGT DNA and extrachromosomal genes, often as part of plasmids as large as 1 Mb in size. These plasmids are particularly rich in HGT DNA (up to 50%), contain a much higher density of mobility genes and pseudogenes, unique species‐specific genes and niche‐adaptive determinants, specifically catabolic (McLeod *et al.*, 2006; Letek *et al.*, 2010). The metabolic complexity of the environmental *Rhodococcus* spp. is a likely reflection of the isolation criteria, seeking for specific abilities such as degradation of multiple aromatic pollutants, biotransformation or production of secondary metabolites (van der Geize and Dijkhuizen, 2004; Larkin *et al.*, 2005; Yamashita *et al.*, 2007; Kitagawa and Tamura, 2008; Holder *et al.*, 2011; Foster *et al.*, 2014). For example, compared to *R. equi*, the polychlorinated biphenyl degrader *R. jostii* RHA1 contains a much larger complement of unique metabolic genes, aromatic gene clusters (29 vs only three), non‐ribosomal peptide synthases (24 vs 11) and polyketide synthases (7 vs 1 in *R. equi*) (McLeod *et al.*, 2006; Letek *et al.*, 2010).

## Circular and linear genomes: a matter of size

The determination of the complete 103S genome sequence made it apparent that *Rhodococcus* spp. differ in chromosome topology despite being monophyletic. While *R. equi* 103S and *R. erythropolis* PR4 both possess covalently closed circular chromosomes, *R. jostii* RHA1 and *R. opacus* B4 have linear ones. Remarkably, not only the four species belong to a same subdivision of the genus *Rhodococcus*, but *R. erythropolis* and *R. jostii*/*R. opacus* even belong to sister sublineages within the same terminal clade (no. 2, see below Fig. 5) (Anastasi *et al.*, 2016). Since the four chromosomes share the same overall structure and synteny (Letek *et al.*, 2010; Anastasi *et al.*, 2016), the only obvious difference is a comparatively larger size for *R. jostii* and *R. opacus* (≥7.25 Mb), similar to *Streptomyces* spp (≥8 Mb), which also possess linear genomes. Thus, actinobacterial chromosome linearization appears to occur as a function of increasing size rather than phylogenetic background. This mirrors the situation with the rhodococcal plasmids, which independently of the host species tend to be linear above 100 kb (Larkin *et al.*, 2010; Valero‐Rello *et al.*, 2015).

## Plasmid‐determined virulence

**Figure 3:**
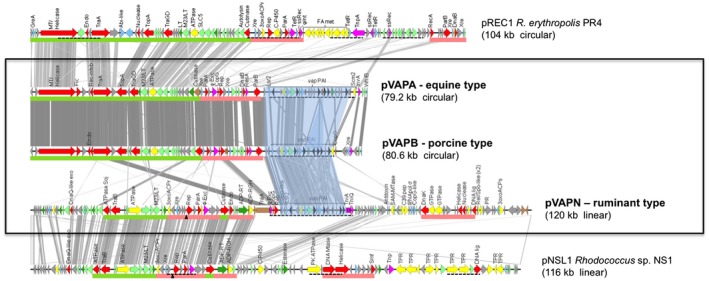
The three host‐specific *R. equi* virulence plasmids. Comparison of pVAPA (equine type) and pVAPB (porcine type) circular virulence plasmids and the recently characterized linear pVAPN plasmid (ruminant type) with closest homologs from environmental biodegrader *Rhodococcus* spp. Regions of significant similarity are connected with grey stripes. The *vap* PAIs are shaded in light blue. Gene colour code: Hypothetical proteins (gray), conjugation or DNA replication/recombination/metabolism (red), DNA mobility genes (magenta), transcriptional regulators (blue), secreted proteins (dark green), membrane proteins (pale green), metabolic functions (yellow), *vap* family genes (black) and pseudogenes (brown).Green and pale red bars below the genes indicate conjugation and replication/partitioning functional modules respectively; dashed underline indicates HGT region. Modified from Valero‐Rello *et al.* (2015)

A distinguishing feature of the genus *Rhodococcus* is the characteristic presence of large circular or linear conjugative plasmids carrying niche‐adaptive DNA (Larkin *et al.*, 2010). While these regions encode catabolic and detoxification pathways in rhodococcal species isolated from xenobiotic‐contaminated ecosystems (McLeod *et al.*, 2006; Sekine *et al.*, 2006), in the pathogenic species *R. equi* and *R. fascians* they encode virulence (i.e. host‐adaptive) determinants (Letek *et al.*, 2008; Francis *et al.*, 2012; Valero‐Rello *et al.*, 2015). In the case of *R. equi*, the plasmid's HGT‐acquired *vap* PAI (Fig. 3) supports intramacrophage survival and is essential for animal host colonization (Coulson *et al.*, 2010).

**Figure 4:**
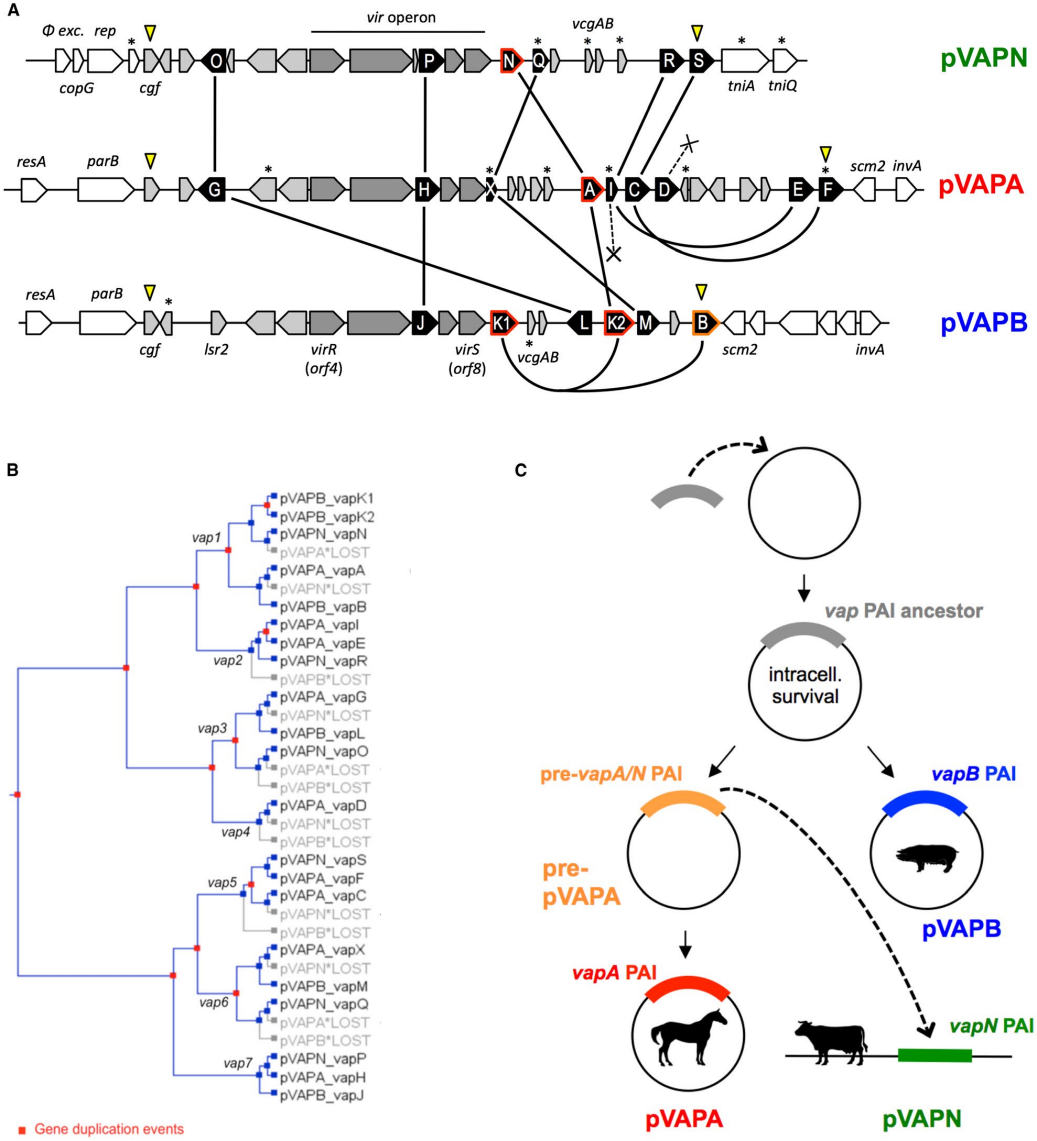
Structure and evolution of the host‐specific *vap* PAIs. Modified from Valero‐Rello *et al.* (2015). A. Genetic structure of the *vap* PAIs from pVAPA (15.1 kb), pVAPB (21.5 kb) and pVAPN (15.9 kb). PAI genes in grey (non‐*vap* genes, in darker shade the *vir* operon) or black (*vap* genes). Genes outside the PAIs in white. PAI boundaries indicated by yellow arrowheads. The figure schematizes the evolutionary relationships of the *vap* genes as inferred from phylogenetic analysis, gene duplication/loss analysis (panel B) and genetic structure comparison. Straight lines connect allelic variants of same *vap* gene ancestor; those of *vapA* have red surround, curved lines indicate *vap* gene duplications. Crosses denote *vap* genes that were lost. Asterisks indicate pseudogenes. B. Gene duplication and loss in *R. equi vap* multigene family. Constructed with notung v2.6 from a *vap* gene ML tree. The analysis indicates that the common ancestor of the three host‐specific PAIs contained seven *vap* genes which evolved by gene duplication from a single ancestor *vap* gene. C. Fate of the *vap* PAI during host‐driven *R. equi* virulence plasmid evolution.

The *vap* PAI encodes a set of homologous secreted virulence‐associated proteins (Vap) (Letek *et al.*, 2008; Valero‐Rello *et al.*, 2015) (Fig. 4A) that fold in a cork‐shaped eight‐stranded antiparallel β‐barrel structure (Geerds *et al.*, 2014; Whittingham *et al.*, 2014). One of them, designated VapA in the equine‐type plasmid pVAPA, is essential for pathogenesis (Jain *et al.*, 2003; Gonzalez‐Iglesias *et al.*, 2014; Valero‐Rello *et al.*, 2015). The exact mechanism of action of VapA and homologous proteins remains unknown but is thought to be related to the biogenesis of the modified, Rab7‐positive endosome where the bacterium replicates within macrophages (aka the “*R. equi*‐containing vacuole’’, RCV) (Fernandez‐Mora *et al.*, 2005; Sydor *et al.*, 2013; Rofe *et al.*, 2017). Consistent with this, VapA has recently been found to localize to the membrane of the RCV (Wright *et al.*, 2018). Non‐*vap* genes are also present in the PAI (Valero‐Rello *et al.*, 2015; MacArthur *et al.*, 2017), notably the *vir* operon, which encodes two key regulators (VirR and VirS) required for *vap* PAI gene activation and virulence (Byrne *et al.*, 2007) (Fig. 4A). Another *vir* operon product, IcgA, has been shown to modulate intracellular growth of *R. equi* (Wang *et al.*, 2014)*.*

A number of core chromosomal metabolic genes appear to have been co‐opted within the regulatory network of the *vap* PAI and exhibit expression patterns similar to those of the plasmid virulence genes. Two of these genes, encoding chorismate mutase and anthranilate synthase enzymes involved in aromatic amino acid biosynthesis, appear to facilitate intracellular survival in macrophages (Letek *et al.*, 2010).

## Plasmid‐mediated host tropism: a novel paradigm

Three *R. equi* virulence plasmid types have been identified to date, pVAPA and pVAPB associated with equine and porcine isolates, respectively, and pVAPN (‘N’ for no‐A/B) associated with ruminants (bovines, ovines and caprines) (Ocampo‐Sosa *et al.*, 2007). pVAPA and pVAPB are variants of a same circular replicon which differ in *vap* PAI structure (Letek *et al.*, 2008), whereas pVAPN is an unrelated linear plasmid, with again a specific *vap* PAI (Valero‐Rello *et al.*, 2015; MacArthur *et al.*, 2017) (Fig. 3). pVAPA/B/N type‐host mismatch virtually never occurs among equine, porcine and ruminant isolates, suggesting stringent host‐driven exclusion of non‐adapted plasmids (Ocampo‐Sosa *et al.*, 2007; unpublished data from JV‐B laboratory). Phylogenomic analyses did not find any association between host and chromosomal genotype but, instead, clear evidence of active exchange of the pVAPA/B/N plasmids across the *R. equi* population, with corresponding host jumps (Anastasi *et al.*, 2016; MacArthur *et al.*, 2017). The *R. equi* virulence plasmid appears to be easily lost in the absence of host selection (Takai *et al.*, 1994; Ocampo‐Sosa *et al.*, 2007) but can be readily reacquired via conjugation (Tripathi *et al.*, 2012; Valero‐Rello *et al.*, 2015). The available evidence supports a model whereby *R. equi* species‐specific infectivity is mediated by the virulence plasmids, with dynamic plasmid loss‐regain allowing flexible adaptation to saprotrophic life in the environment and parasitization of different animal hosts.

Contrasting with their selectivity for certain animal species, the three host‐adapted plasmids are commonly found in human isolates (Ocampo‐Sosa *et al.*, 2007; Anastasi *et al.*, 2016). This suggests that animals are the source of infection for people, establishing *R. equi* as a novel zoonotic pathogen. It also implies that humans are essentially opportunistic hosts for *R. equi* (Ocampo‐Sosa *et al.*, 2007; Vazquez‐Boland *et al*., 2013, 2010). The situation appears to be analogous for other animal species which seem to be also accidental hosts for *R. equi*, as for example suggested by recent virulence plasmid characterization studies from dog isolates (Bryan *et al.*, 2017).

## Evolution of *R. equi* virulence

As mentioned above, the pVAPA/B/N plasmids each carry a type‐specific *vap* PAI. The major differences lie in the *vap* multigene family (Fig. 4A). Phylogenetic reconstruction of *vap* multigene family evolution indicates that the nearest common ancestor of the *vap* PAI contained seven *vap* genes (Valero‐Rello *et al.*, 2015). These progenitor *vap* alleles originated via gene duplication from an ancestor *vap* determinant (Fig. 4B). This proto‐*vap* gene was probably horizontally acquired because obvious homologs are absent from other *Actinobacteria* while they are found in bacteria from different phyla and even fungi, yet remaining relatively uncommon (Whittingham *et al.*, 2014; Valero‐Rello *et al.*, 2015).

A likely hypothetical scenario is that the proto‐*vap*, in combination with some non‐*vap* determinant present in the common PAI ancestor, acquired at some stage the ability to promote intracellular survival. Perhaps initially a defence mechanism against predation by bacterivore environmental protozoa, eventually this also allowed the host bacterium to escape phagocytic killing by macrophages, paving the way to becoming an animal pathogen. Indeed, a critical intracellular survival determinant is obviously present in the extant *vap* PAIs, because the three host‐adapted plasmids promote intracellular survival in cultured macrophages (Giguere *et al.*, 1999; Coulson *et al.*, 2010; Gonzalez‐Iglesias *et al.*, 2014; Valero‐Rello *et al.*, 2015; Willingham‐Lane *et al.*, 2016).

Cumulative epidemiological and experimental evidence indicates that the intracellular survival‐promoting function is primordial and dissociable from host tropism, because the three plasmid types promote virulence in accidental (non‐adapted) animal hosts (e.g. mice, apparently also humans). This critical *vap* determinant is probably the common ancestor of *vapA* of the equine pVAPA type and its allelic variants *vapN* of the ruminant pVAPN type (Valero‐Rello *et al.*, 2015) and *vapK1/2* (and putative duplicate thereof, *vapB*) of the porcine pVAPB type (Valero‐Rello *et al.*, 2015; Willingham‐Lane *et al.*, 2018) (Fig. 4A and B).

Subsequently, host‐tropic properties evolved in the common ancestor of the *vap* PAI, presumably through adaptive evolution of the *vap* multigene family in equines, swines and ruminants. The process appears to have started in the pre‐pVAPA/B plasmid, followed by horizontal transfer of the PAI from the pVAPA lineage to the pVAPN replicon (Valero‐Rello *et al.*, 2015) (Fig. 4C). The perfect conservation of the *vap* PAIs within each host‐adapted virulence plasmid type indicates they are under strong selection, likely driven by species‐specific host factors yet to be identified. The conservation of the DNA mobility gene remnants flanking the *vap* PAIs and the pseudogenes in each PAI type suggests that the PAI diversification process is relatively recent (MacArthur *et al.*, 2017).

## Common rhodococcal strategy for rapid niche adaptation

The *R. equi* virulence plasmids share similar backbones with other plasmids found in environmental rhodococci. Thus, the pVAPA/B replicon is homologous to that of pREC1 from the alkane degrader *R. erythropolis* PR4 (Sekine *et al.*, 2006) or pKNR from the organic solvent‐tolerant *R. opacus* B4 (Honda *et al.*, 2012) (Fig. 3). All these circular plasmids possess a conjugation machinery based on a MOBf (TrwC)‐type relaxase (Garcillan‐Barcia *et al.*, 2009), designated TraA, together with a type IV secretion system (T4SS) which forms the transport channel. pVAPN, on the contrary, is closely related to the linear plasmid pNSL1 from the environmental *Rhodococcus* sp. NS1 (Zhu *et al.*, 2010) (Fig. 3). Like the circular plasmids, pVAPN and pNSL1 share a conserved backbone but differ in a unique variable region (VR) adjacent to the replication/partitioning region (Fig. 3). Self‐transmissibility relies in this case on a relaxase/T4SS‐independent mechanism mediated by a TraB translocase, a novel conjugation system first characterized in the *Streptomyces* linear plasmids. The TraB protein is evolutionarily related to FtsK/SpoIIIE involved in chromosome segregation (Guglielmini *et al.*, 2013) and forms a hexameric channel through which dsDNA is conducted in an ATP‐dependent manner (Vogelmann *et al.*, 2011). While not obviously similar, the pVAPN and pNSL1 replicons are phylogenetically related to other rhodococcal linear plasmids (Valero‐Rello *et al.*, 2015).

The VRs of all the rhodococcal plasmids, whether circular or linear, are typically flanked or contain DNA mobility gene remnants including a variety of recombinases and transposases (Letek *et al.*, 2008; Valero‐Rello *et al.*, 2015) (Fig. 4A). Integrative elements thus appear to play a key role in the formation and plasticity of the VRs. An intriguing feature is the conservation of the *rep*‐*parA* module across the pVAPA/B and pREC1 circular plasmids and the pVAPN and pNSL1 linear plasmids (Fig. 3). The *rep*‐*parA* module is detected as HGT DNA in pREC1 and pNSL1 and adjacent to it there is a putative phage excisionase gene that is also conserved in the circular and linear plasmids. This suggests that the *rep‐parA* module adjoining the VRs itself forms part of a gene cassette that is horizontally exchangeable between different rhodococcal replicons despite their different ancestry (Letek *et al.*, 2008; Valero‐Rello *et al.*, 2015).

The *R. equi* virulence plasmids consolidate the notion that rapid niche adaptation through shared sets of self‐transferable extrachromosomal replicons is a key common attribute of the actinobacterial genus *Rhodococcus*.

## The lingering problem of *R. equi* taxonomy and nomenclature

Although sharing obvious physiological, compositional and genetic features with the other rhodococci, specifically the common plasmid‐driven niche specialization strategy, the taxonomic status of *R. equi* within the genus *Rhodococcus* has been repeatedly questioned (McMinn *et al.*, 2000; Jones and Goodfellow, 2012). These taxonomic difficulties are mirrored in the nomenclature of the species. To several previous validly published names, i.e. *Corynebacterium equi* Magnusson, 1923, *Nocardia restricta* (Turfitt 1944) McClung 1974 and *R. equi* (Magnusson, 1923) Goodfellow and Alderson 1977, additional names have been recently added in rapid succession, as discussed below.

At the root of the nomenclatural instability are a number of 16S rRNA phylogenetic studies, which placed *R. equi* at the periphery of the genus *Rhodococcus* (McMinn *et al.*, 2000; Gurtler *et al.*, 2004) or among the *Nocardia* (Rainey *et al.*, 1995; Goodfellow *et al.*, 1998). Their significance was, however, unclear because of the low bootstrap values (Rainey *et al.*, 1995), or because *Nocardia* branched off from within the *Rhodococcus* radiation (Rainey *et al.*, 1995; Goodfellow *et al.*, 1998; McMinn *et al.*, 2000) instead of forming a deep monophyletic lineage near the base of the *Corynebacteriales* (Ludwig *et al.*, 2012).

A recent publication by Jones *et al.* (2013a) based on numerical phenetic and genotypic (PCR fingerprinting, 16S rDNA sequence) clustering further complicated the situation. While, as expected from their taxonomic status as a separate species, *R. equi* strains grouped in a distinct cluster, perfectly equivalent to that formed by other rhodococcal clades, this fact was used to justify the reclassification of *R. equi* into a new genus, as ‘*Prescottia equi*’ gen. nov., comb. nov. (Jones *et al.*, 2013a). The name ‘*Prescottia*’ was proposed in honour of the *R. equi* research pioneer John F. Prescott from University of Guelph in Canada. However, the new genus name was found to be illegitimate according to the International Code of Nomenclature of Prokaryotes (aka the Bacteriological Code) (Lapage *et al.*, 1992) because it already designated the *Orchidaceae* plant genus *Prescottia* Lindley 1824. The same authors corrected the mistake by proposing ‘*Prescotella*’ gen. nov., and ‘*Prescotella equi*’ comb. nov. as its sole species (Jones *et al.*, 2013b).

The proposal to transfer *R. equi* to a new genus was presented to the international *R. equi* community at the 5th Havemeyer Workshop on *R. equi* held in Deauville (France) on 9–12 July 2012 and was largely met with disapproval for several reasons (Cauchard *et al.*, 2013). First, it clashed with the conclusions of the recently published 103S genome study, which found that *R. equi* was genomically well classified as a *Rhodococcus* sp. (Letek *et al.*, 2010). Second, strong concerns were voiced that changing such a well‐consolidated name in veterinary and medical science would cause considerable confusion. Another important caveat was whether the methodology used in the Jones *et al.* (2013a) study had adequate resolution to define taxa above the species level according to modern phylotaxonomic criteria. The opinion was expressed that reassigning *R. equi* to a novel genus would only be justified upon systematic re‐examination of the taxonomy of the entire genus *Rhodococcus* using phylogenomic approaches (Cauchard *et al.*, 2013). We transmitted these concerns on behalf of the international *R. equi* community to leading members of the International Committee for Systematics of Prokaryotes (ICSP) in correspondence exchanges in March 2013.

Probably due to the stir caused by the proposed name changes, the nomenclature of the species was re‐examined and it became apparent that *R. equi* had an earlier heterotypic synonym in *Corynebacterium hoagii* (Morse 1912) Eberson 1918 (Tindall, 2014a). Evidence for the identity of *R. equi* and the nomenclatural type *C. hoagii* as the same species was provided by 16S rRNA gene phylogenies used in the description of the closely related species *R. defluvii* (Kämpfer *et al.*, 2014). This meant that not only ‘*Prescotella equi*’ was an illegitimate name but that the name *R. equi* also contravened itself the principle of priority of the Bacteriological Code (Tindall, 2014a). Since it was evident that *R. equi* is not a *Corynebacterium*, and Kämpfer *et al.* (2014) confirmed that the differentiation of *R. equi* from other members of the genus *Rhodococcus* was unsupported by chemotaxonomic and phylogenetic evidence, these authors proposed to retain *R. equi* within the genus *Rhodococcus* but with the priority epithet *hoagii*, as *Rhodococcus hoagii* comb. nov.

Truly, a ‘fine mess’, to quote the authors who prompted this ‘nomenclatural storm’ by proposing the new genus names ‘*Prescottia*/*Prescottella*’ (Goodfellow *et al.*, 2015).

## *R. equi* is genomically a *bona fide Rhodococcus*

**Figure 5:**
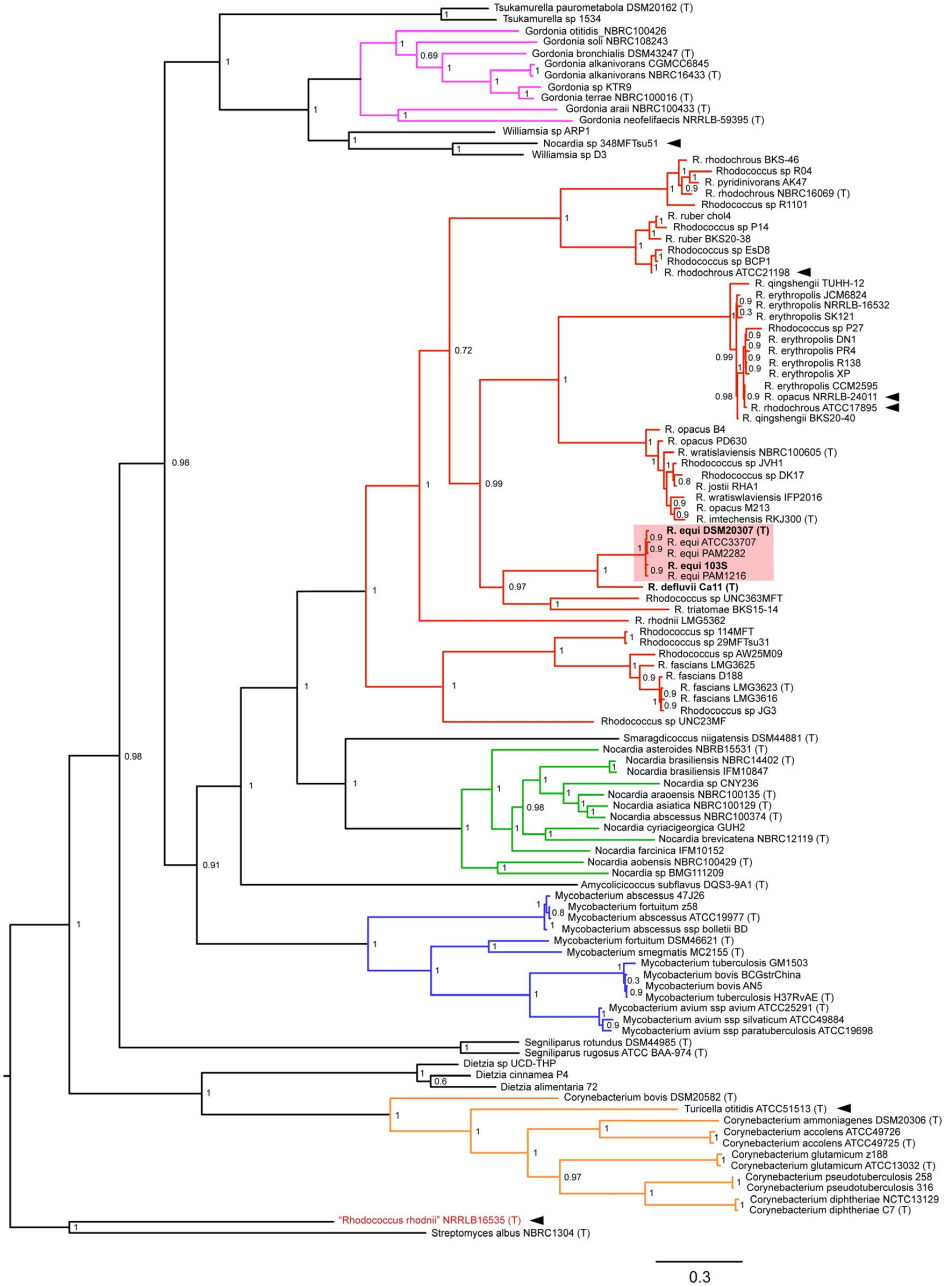
Whole‐genome *Corynebacteriales* ML tree. Nodes indicate bootstrap values. Tree constructed with five *R.equi* genomes, 47 non‐*equi Rhodococus* genomes including representatives from the major 16S rRNA gene clades (Goodfellow *et al.*, 1998; McMinn *et al.*, 2000; Jones and Goodfellow, 2012; Ludwig *et al.*, 2012), and 57 genomes from 11 *Corynebacteriales* genera. Rooted with *Streptomyces albus* NBRC 1304^T^ (outgroup). (T) indicates type strain. Genome used for *R. equi* type strain DSM 20307^T^ = ATCC 6939^T^ is assembly acc. no. LWTX00000000 (Anastasi *et al*., 2016). Major genera are highlighted in different colour. Black arrowheads indicate misclassifications revealed by the phylogenomic analysis. One of them is *R. rhodnii* NRRL B‐16535^T^ (GenBank assembly acc. no. GCA_000720375.1); this probably represents a sequence mislabelling or strain mixup. Modified from Anastasi *et al.* (2016).

Anastasi *et al.* (2016) performed a detailed whole genome sequencing (WGS) phylogenetic analysis of a collection of representative *R. equi* isolates, including the type strain DSM 20307^T^ (= ATCC 6939^T^), and available sequences from other rhodococcal and *Corynebacteriales* species (Fig. 5). An important first conclusion is that *R. equi* is a strictly monomorphic taxon (Fig. 2), thus settling the question of whether *R. equi* is phylogenetically heterogeneous. This had been often raised in the literature (McMinn *et al.*, 2000; Gurtler *et al.*, 2004; Jones and Goodfellow, 2012), curiously based on just a single 16S rDNA study that reported similarity values of 97.9–98.3% between the *R. equi*‐type strain ATCC 6939^T^/ DSM 20307^T^ and some of the (only 10) isolates analysed (McMinn *et al.*, 2000). The reason for such rRNA sequence variability is unclear, but might be due to possible strain misidentifications (Vazquez‐Boland *et al.*, 2010) and/or 16S rDNA sequencing errors. In the genomes analysed by Anastasi *et al.* (2016), representing a diversity of genetic backgrounds and isolation sources, all 16S rDNA sequences were 100% identical.

Secondly, the phylogenomic studies disambiguated the taxonomic relationship of *R. equi* with other *Rhodococcus* spp. All *R. equi* isolates group in a well‐supported monophyletic cluster (no. 3 or ‘*equi*’ subclade) deeply rooted in the rhodococcal phylogeny which also contains *R. defluvii* Ca11^T^s as the closest relative, *Rhodococcus triatomae* BKS15‐14 (a species not previously resolved into any specific 16S rRNA gene clade) (Ludwig *et al.*, 2012), and an unclassified isolate (Anastasi *et al.*, 2016) (Fig. 5). Two other subclades are generally congruous with the 16S rDNA groupings ‘*rhodochrous*’ (subclade 1) and ‘*erythropolis*’ (subclade 2) (McMinn *et al.*, 2000; Jones and Goodfellow, 2012; Ludwig *et al.*, 2012). Subclade 1 splits into two distinct sublineages, one encompassing *R. ruber*, and the other, the type species of the genus, *R. rhodochrous* and *Rhodococcus pyridinivorans*. Subclade 2 also splits into two sublineages, one containing *R. opacus*, *R. jostii*, *Rhodococcus imtechensis* and *Rhodococcus wratislaviensis*; the other, *R. erythropolis* and *Rhodococcus qingshengii* (Fig. 5). The remaining major phyletic lines correspond to *R. rhodnii* LMG 5362 and *R. fascians* isolates, defining two novel subclades (nos. 4 and 5 respectively). Subclade 5 ‘*fascians*’ branches off at an early bifurcation in the *Rhodococcus* phylogeny (Anastasi *et al.*, 2016) (Fig. 5).

The general conclusion of these WGS studies is that *R. equi* is a prototypic *Rhodococcus*, and that if a new genus were to be created to accommodate the species, the same treatment would need to be applied for each of the major rhodococcal lineages (Anastasi *et al.*, 2016). Such an atomization of the genus seems unjustified because *Rhodococcus* forms a distinct and biologically coherent and uniform monophyletic grouping comparable in rank and diversity to other well‐established *Corynebacteriales* genera, such *Corynebacterium*, *Gordonia* or *Mycobacterium* (Anastasi *et al.*, 2016) (Fig. 5). It would also defeat the very purpose of bacterial nomenclature in facilitating the coherent study of evolutionarily and biologically related organisms assembled under a common taxon name.

## *Corynebacteriales* phylogenomics

The phylogenomic analyses by Anastasi *et al.* (2016) also clarified the evolutionary relationships of the rhodococci with other *Corynebacteriales*, in particular *Nocardia*, inconsistently resolved by previous 16S rDNA phylogenies (Rainey *et al.*, 1995; Goodfellow *et al.*, 1998; McMinn *et al.*, 2000; Jones and Goodfellow, 2012; Ludwig *et al.*, 2012; Kämpfer *et al.*, 2014). *Rhodococcus* and *Nocardia* form two clearly distinct clades within a well‐supported phyletic line that also comprises *Smaragdicoccus niigatensis* DSM44881^T^, classified in the *Nocardiaceae* (as is *Rhodococcus*), as well as *Mycobacterium* spp. and *Amycolicicoccus subflavus* DQS3‐9A1^T^, a single‐species genus of the *Mycobacteriaceae* (Fig. 5). Another major *Corynebacteriales* line of descent is formed by members of the genera *Gordonia* and *Williamsia*, classified in the *Nocardiaceae*, and *Tsukamurella* of the monogeneric *Tsukamurellaceae* (Fig. 5). Two additional major lines of descent are clearly defined, one encompassing *Corynebacterium*, *Turicella otitidis* ATCC15513^T^ (*Corynebacteriaceae*) and *Dietzia* (family *Dietziaceae*), the other corresponding to *Segniliparus* spp. (family *Segniliparaceae*) (Anastasi *et al.*, 2016) (Fig. 5).

The phylogenomic data, therefore, indicate that the current *Nocardiaceae* taxon is polyphyletic and call for a possible reclassification of the *Corynebacteriales* into four families: a) *Mycobacteriaceae*, including the genera *Mycobacterium*, *Amycolicicoccus*, *Smaragdicoccus*, *Rhodococcus* and *Nocardia*; b) *Gordoniaceae*, with the genera *Gordonia*, *Williamsia* and *Tsukamurella*; c) *Corynebacteriaceae*, with the genera *Corynebacterium*, *Turicella* and *Dietzia*; and d) *Segniliparaceae*.

## **Conservation of *Rhodococcus equi* (Magnusson 1923) Goodfellow and Alderson 1977 and rejection of *Rhodococcus hoagii* (Morse 1912) **Kämpfer *et al.* 2014

While, as discussed above, there appears to be no reasonable grounds for transferring *R. equi* to a new genus, the problem remains with the epithet *hoagii*. Though valid and legitimate in strict nomenclatural terms, the name *R. hoagii* is met with rejection in the context where the organism is relevant. The name *R. equi* is very well established, widely accepted and in widespread use, not only among the veterinary community and equine industry but also the medical profession where the bacterium is recognised as a human opportunistic pathogen. The epithet *equi* suitably encapsulates the very essence of *R. equi* and its significance for the communities concerned and the public. In contrast, the *hoagii* epithet has remained largely in disuse, restricted to an obscure type strain characterized by features such as production of oxoalkylxanthines and pregnadienes, with no obvious connection with the identity of *R. equi* as a well‐known pathogen.

Apart from the confusion already generated due to the use of the new epithet *hoagii* in gene repositories and genomic databases, the application of *R. hoagii* is likely to cause significant problems of traceability and interpretation of the literature. We believe that this falls under the concept of *nomen perplexum*, one of the exceptions allowing rejection of a bacterial name (Rule 56a.4 of the Bacterial Code, perplexing name: ‘a name whose application is known but which causes uncertainty in bacteriology’) (Lapage *et al.*, 1992). This situation would be definitely compounded if in addition the genus name were to be changed (see below).

Where there is no doubt that the epithet *hoagii* meets the provisions for rejection is under Rule 56a.5 *nomen periculosum*, ‘a name whose application is likely to lead to accidents endangering health or life or both or of serious economic consequences’ (Lapage *et al.*, 1992). We cannot think of a more accurate adherence to the notion of a perilous name. There are real chances of potential misdiagnoses or inaccurate risk appraisals due to confusion generated as a result of the introduction of the name *R. hoagii* to designate a pathogenic microbe with a previously well consolidated and recognized name.

The above points have been already evoked by Garrity (2014) in his Request for an Opinion (RfO) to the Judicial Commission of the ICSP for conservation of *R. equi* as the valid and legitimate name for the taxon. We entirely align ourselves with the views expressed by our colleague. However, Garrity's RfO was formulated to reject the name *Corynebacterium hoagii* and since then the name *Rhodococcus hoagii* has been validated by inclusion in a Notification List of approved names (Oren and Garrity, 2014). A formal request – which we hereby are formulating – is therefore needed for the conservation of *Rhodococcus equi* (Magnusson, 1923) Goodfellow and Alderson 1977 and rejection of *Rhodococcus hoagii* (Morse 1912) Kämpfer et al. 2014.

## Concluding remarks

The same arguments as above apply for the reassignment of the species to a new genus ‘*Prescotella*’, still advocated by its proposers (Goodfellow *et al.*, 2015) despite its evident biological inadequacy, the confusion that it will create, the rejection by the *R. equi* community (Cauchard *et al.*, 2013), and the implications for the nomenclature of the whole genus *Rhodococcus* (Letek *et al.*, 2010; Anastasi *et al.*, 2016).

Probably as a side effect of the nomenclatural debate stemming from the proposed reclassification of *R. equi* into a new genus ‘*Prescottia/Prescotella*’ which turned out to be illegitimate, a significant potential new difficulty arose with the realization that *Rhodococcus* Zopf 1891 may be illegitimate itself, because a later homonym of the algal genus *Rhodococcus* Hansgirg 1884 (Tindall, 2014b). Can one reasonably conceive changing well‐established bacterial names such as *Staphylococcus*, *Escherichia* or *Salmonella*? The situation with *R. equi* is essentially analogous. The bacterial genus *Rhodococcus* Zopf 1891 has a comparable standing in the scientific literature and a potential change may have disastrous consequences. The same applies to the name *R. equi* in veterinary and medical microbiology. This extends to the terminology of the infectious disease itself: ‘rhodococcal pneumonia’, ‘rhodococcal infection’ and ‘foal rhodococcosis’ are all well established and commonly used terms in the veterinary, medical and professional literature.

The phylogenomic confirmation of *R. equi* as a *bona fide Rhodococus* contributes to the picture of a highly adaptable genus of *Actinobacteria* with a diversity of lifestyles, from saprotrophic biodegraders to plant pathogens and animal intracellular parasites. Further illustrating the unique versatility of this group of bacteria, the major *Rhodococcus* phyletic line within which *R. equi* evolved contains species with both circular and linear chromosomes. The genus *Rhodococcus* as it currently stands, including *R. equi*, thus serves to consolidate the important notion that genome topology is primarily a consequence of genome size and has no intrinsic taxonomic value. Together with the shared plasmid‐driven niche‐adaptive strategy, it showcases the extraordinary flexibility of the bacterial genome to ensure rapid accommodation to different ecological scenarios.
